# Shrimp shell chitosan improves preservation quality and *in vitro* rumen digestibility and reduces methane concentration

**DOI:** 10.5455/javar.2026.m1045

**Published:** 2026-06-22

**Authors:** Rakhmad Perkasa Harahap, Yeti Rohayeti, Saitul Fakhri, Anuraga Jayanegara, Sri Suharti, Nahrowi Nahrowi

**Affiliations:** 1 Study Program of Animal Science, Faculty of Agriculture, Tanjungpura University, Jalan Prof. Dr. H. Hadari Nawawi, Pontianak 78124, Indonesia; 2 Department of Nutrition and Feed Science, Faculty of Animal Husbandry, Jambi University, Jl. Raya Jambi–Muara Bulian Km.15, Jambi 36361, Indonesia; 3 Department of Animal Nutrition and Feed Technology, Faculty of Animal Science, IPB University, Jl. Agatis, Kampus IPB Dramaga, Bogor 16680, Indonesia

**Keywords:** Chitosan, *in vitro*, methane concentration, rumen, shrimp shell-derived, total mixed ration

## Abstract

**Objectives:** This research assessed the impact of additives derived from shrimp shells, specifically chitosan, on the preservation of total mixed rations (TMR) in both anaerobic and aerobic environments, along with their effects on *in vitro* rumen digestibility, fermentation attributes, and methane production.

**Materials and Methods:** A 2 × 4 factorial randomized complete block design was employed, featuring five replications. The study included two preservation conditions (anaerobic and aerobic) and four additive treatments: control, shrimp shell, chitin, and chitosan at 1% of fresh matter. After 30 days of storage, we assessed the preservation characteristics and the *in vitro* rumen fermentation variables.

**Results:** Anaerobic preservation led to decreased dry matter loss, pH, and NH_3_, and to increased total volatile fatty acids compared with aerobic preservation (*p* < 0.01). Chitosan showed the most beneficial preservation effect, exhibiting the least dry matter loss (3.50%) and pH (5.20), along with the highest organoleptic quality. Significant interactions between treatment and additive were observed for traits related to preservation, suggesting that the positive effects of chitosan were more pronounced under anaerobic conditions. Additionally, chitosan achieved the highest *in vitro* dry matter and organic matter digestibility (56.61% and 58.30%, respectively), along with the lowest methane concentration and cumulative gas production (*p* < 0.05).

**Conclusions:** Shrimp shell-derived chitosan at 1% fresh matter effectively enhances TMR preservation and improves rumen fermentation, thereby increasing digestibility and reducing methane concentration. This indicates the potential to use shrimp shell waste as a valuable feed additive, promoting sustainable ruminant feeding and supporting circular economy practices.

## 1. Introduction

Total Mixed Ration (TMR) is a strategy for feeding livestock that combines all feed components into a single mixture, ensuring a well-balanced diet while minimizing selective feeding behaviors. TMR anaerobic preservation generally maintains levels of crude protein (CP), neutral detergent fiber (NDF), and non-fibrous carbohydrates. However, the concentrations of soluble CP and sugars can vary during the fermentation process [1]. The presence of oxygen triggers the activity of aerobic microorganisms, such as yeasts and molds, which deplete nutrients in the anaerobic preservation, resulting in increased dry matter (DM) losses and diminished nutritional value [2]. Additionally, aerobic spoilage promotes proteolysis, as evidenced by elevated ammonia-N levels [3]. Therefore, it is crucial to apply additives for effective anaerobic preservation.

Among natural additives, chitosan has attracted increasing attention due to its antimicrobial and biofunctional properties. Additives derived from shrimp shells offer a unique, largely unexplored opportunity, combining preservation capabilities with the potential to influence rumen fermentation. Chitin and chitosan, extracted from shrimp shell byproducts, are versatile biopolymers with significant promise as natural additives due to their combined antimicrobial, antioxidant, and other biofunctional properties. Chitosan exhibits a broad range of antimicrobial effects against bacteria, yeasts, and molds, helping inhibit spoilage microorganisms and contributing to preservation stability [4, 5, 6]. Additionally, its antioxidant properties can reduce oxidative damage and extend shelf life, thereby enhancing antimicrobial protection by mitigating quality losses associated with oxygen exposure [5, 6]. Furthermore, incorporating chitosan into ruminant diets has demonstrated its ability to modify rumen fermentation patterns. It boosts propionate production while lowering acetate and butyrate levels, thereby enhancing feed efficiency and reducing methane concentration [7, 8, 9]. Both chitin and chitosan possess antimicrobial and biofunctional properties that may restrict aerobic spoilage organisms and proteolysis during the ensiling process, while also influencing rumen microbial activity after feeding.

Recent research has shown that chitosan can improve rumen fermentation, alter microbial communities, and, in some cases, lower methane production. Nevertheless, the existing evidence is distributed across various preservation methods and rumen evaluation methods. Most studies on ruminants have focused on chitosan as a dietary supplement, either *in vivo* or *in vitro*, examining factors such as shifts in fermentation, digestibility, nitrogen metabolism, and methane reduction, without accounting for its role in feed preservation [10]. In contrast, the few studies on chitosan in preserved total mixed rations (TMR) have mainly looked at different inclusion rates and its effects on ruminal fermentation and degradability, rather than providing a direct comparison of raw shrimp shell, chitin, and chitosan derived from the same by-product [11]. Thus, there is still a considerable lack of robust evidence on the relative effectiveness of shrimp shell-derived materials throughout the entire process, from both anaerobic and aerobic preservation to subsequent *in vitro* rumen digestibility, fermentation characteristics, and methane outputs. Filling this gap is essential not only to determine if chitosan is more effective than its precursor materials but also to aid in the valorization of shrimp-processing waste as a functional feed additive within a more sustainable and circular livestock production framework [9].

This study examined not only the effects of shrimp shell-derived additives on the preservation quality of anaerobically stored TMR but also their subsequent influence on *in vitro* rumen fermentation profiles. This research aimed to evaluate the impact of shrimp shell-derived additives (including shrimp shell, chitin, and chitosan) on the preservation quality of TMR stored anaerobically and aerobically and to assess their effects on *in vitro* rumen fermentation patterns and methane (CH_4_) concentration.

## 2. Materials and Methods

### 2.1. Ethical approval

The Institutional Review Board (IRB) of the Animal Husbandry Program at Universitas Tanjungpura approved the research protocol for the use of rumen fluid, assigning the approval number IRB-PTN/2025/017. All animal procedures followed the ethical standards of the Institutional Research Committee.

### 2.2. Sample preparation and ensiling procedure

This research investigation utilized additives derived from shrimp shells, such as chitosan (with a degree of deacetylation of 87.5%, viscosity of 50 cps, pH of 7.1, and 99% solubility), chitin (with a degree of deacetylation of 65.3%), and raw shrimp shell (with a degree of deacetylation of 0%). All additives were sourced from the commercial supplier BioChitosan Indonesia. The base total mixed ration (TMR) was composed of various ingredients, assessed on a dry matter basis, including 6.0% cassava pulp, 10.0% rice bran, 13.0% wheat pollard, 4.0% soybean meal, 25.0% palm kernel meal, 12.0% coconut meal, 5.0% molasses, 1.0% salt, 1.5% CaCO_3_, 20.0% coffee hulls, and 2.5% spent tea leaves. The composition and nutrient content of the TMR are detailed in Table 1. The TMR utilized was a commercial formulation from Agro Palacio, a commercial supplier based in Bogor, Indonesia. Proximate analyses were conducted in duplicate following AOAC [12] methodologies. Table 1 presents the TMR formulation utilized in this study, specifically chosen to meet the nutritional requirements of local cattle in Indonesia. It corresponds with a meta-analysis indicating that indigenous cattle breeds in Indonesia have distinct protein and energy needs for maintenance and growth [13]. The diet contains 10.20% crude protein and 64.05% total digestible nutrients on a dry matter basis, both of which are within a practical range to support cattle maintenance and growth.

**Table 1.** Composition and nutrient content of TMR.

Composition	Content
Feed ingredients (% DM)	
Cassava pulp	6.0
Rice bran	10.0
Pollard	13.0
Soybean meal	4.0
Palm kernel meal	25.0
Coconut meal	12.0
Molasses	5.0
Salt	1.0
CaCO_3_	1.5
Coffee hulls	20.0
Tea waste	2.5
Chemical composition (% DM)	
Dry matter (DM)	87.30
Neutral detergent fiber (% of CF)	59.90
Acid detergent fiber (% of CF)	48.60
Crude protein (% of DM)	10.20
Ether extract (% of DM)	6.30
Crude fiber (% of DM)	6.70
Ash (% of DM)	12.60
Total digestible nutrients (% of DM)	64.05

DM = Dry matter, CF = Crude fiber.

The experimental design employed was a randomized complete block design with a 2 × 4 factorial arrangement and five replications (blocks). The first factor pertained to the processing of total mixed rations (TMR), categorized as anaerobically stored TMR (TMR anaerobically stored and incubated for 30 days under anaerobic conditions) and aerobically stored TMR (TMR aerobically stored). The second factor involved the type of shrimp shell-derived additive: no additive (control), shrimp shell, chitin, and chitosan, each at 1% of fresh matter (equivalent to 10 gm/kg of fresh TMR).

The additive dose of 10 gm/kg fresh Total Mixed Ration (TMR), equivalent to 1% of the fresh matter, was chosen based on literature and practical considerations. A study on TMR silage found that chitosan was most effective at enhancing rumen fermentability and nutrient degradability at concentrations of 1.0% to 1.5% [11]. Other preservation studies of alfalfa and corn silage also supported 1% as an effective intermediate dose [14]. This concentration reduces the disadvantages of high inclusion while effectively eliciting measurable biological reactions. The research employed 10 gm/kg of fresh TMR as a baseline dose to compare the shrimp shell-derived additives.

For TMR stored anaerobically, for each treatment, 1,000 gm of fresh TMR was meticulously blended with the specified additive at a 1% concentration. The mixture was packaged into high-density polyethylene (HDPE) bottles with a volume of approximately 1,000 ml, serving as laboratory silos. Each silo was equipped with a gas outlet tube submerged in water to maintain an anaerobic environment by sealing against oxygen ingress. The silos were stored for 30 days. Each block contained all eight treatment combinations, with separate preparation of each treatment unit for subsequent assessment of organoleptic quality score, anaerobic fermentation characteristics, and *in vitro* rumen fermentation profiles.

Following a 30-day incubation period, the stored material was separated into solid and liquid fractions. The anaerobically stored TMR was mixed with distilled water at a 1:7 weight/volume ratio (30 gm to 210 ml) and subsequently filtered to yield filtrate and residue [15]. The solid portion was dehydrated in an oven at 60°C for 48 h, then ground and analyzed for nutritive value analysis and *in vitro* rumen fermentation tests. The liquid portion was used to assess the fermentation characteristics of anaerobically stored TMR.

The preparation of the aerobic-stored TMR followed the same formulation and additive procedures as the anaerobic TMR, except that oxygen was present. Specifically, 1,000 gm of fresh TMR was mixed with additives (control, shrimp shell, chitin, or chitosan) at 1% of the fresh mass. The mixtures were stored in open plastic containers at ambient laboratory conditions (25–28°C) for 30 days, allowing continuous oxygen exposure.

### 2.3. Organoleptic quality and nutritive value analysis

After unsealing the TMR anaerobically stored and collecting the TMR aerobically stored, each sample was spread evenly on a sanitized table in ambient conditions for organoleptic quality assessment. The sensory characteristics were examined through visual observation and olfactory evaluation, including color, scent, texture, and any noticeable fungal growth. A four-point descriptive scoring system was used to standardize the assessment (Table 2), where a score of 1 indicated the best quality (fresh acidic odor, brown color, non-clumping/non-slimy texture, and no mold), and a score of 4 indicated severe deterioration (putrid odor, black color, pronounced clumping with slime formation, and extensive mold across the surface). A total of 20 non-expert panelists completed a structured evaluation form using the defined indicators, and each sample was scored and summarized as an organoleptic quality score [16]. The use of 20 non-expert panelists followed the previously established preserved-feed evaluation methods. It was considered valid for this study because the assessed traits (color, aroma, texture, and visible fungal growth) were evaluated using standardized scoring criteria [16].

**Table 2.** Organoleptic quality scoring criteria for TMR anaerobic-stored and TMR aerobic-stored.

Score	Color	Aroma	Texture	Fungal presence
1	Brown	Fresh acidic	Not clumped and not slimy	None
2	Dark brown	Acidic	Slightly clumped and slightly slimy	Slight
3	Brownish black	Less acidic	Moderately clumped and slimy	Abundant on the surface
4	Black	Putrid/rotten	Heavily clumped and slimy	Abundant on all surfaces

The nutritive value of TMR was determined according to AOAC [12]. The nutritive value of TMR samples was analyzed in duplicate. Dry matter (DM) was determined by oven drying at 105°C for 6 h to constant weight. Crude ash (CA) was determined by ignition in a muffle furnace at 600°C for 2 h, following the AOAC gravimetric ash procedure (AOAC Official Method 942.05). Crude protein (CP) was assessed based on nitrogen (N) content, utilizing a Micro-Kjeldahl apparatus (AOAC Official Method 984.13) and calculated as N × 6.25. Ether extract (EE) was determined by solvent extraction (AOAC Official Method 920.39), and crude fiber (CF) content was determined by sequential extraction with 0.255 N H_2_SO_4_ and 0.313 N NaOH, followed by an additional 40-min extraction step (AOAC Official Method 978.10).

Neutral detergent fiber (NDF) and acid detergent fiber (ADF) were analyzed following the method described by Van Soest et al. [17]. In brief, 1 gm of the sample was placed in a 600 ml beaker, combined with 100 ml of neutral detergent solution, and heated to boiling for 60 min. The residue was then recovered by filtration and recorded as NDF. ADF was determined using the same procedure, substituting the neutral detergent solution with the acid detergent solution. Both NDF and ADF values were corrected for residual ash. Furthermore, CP was measured in NDF and ADF residues to compute neutral detergent insoluble crude protein (NDICP) and acid detergent insoluble crude protein (ADICP), respectively, as described by Jayanegara et al. [18]. All chemical analyses were carried out in duplicate to ensure their reliability.

### 2.4. TMR anaerobic and aerobic stored fermentation characteristics analysis

TMR anaerobic-stored pH was assessed using a calibrated pH meter (Jenway Model 3505) with pH 7.0 and pH 4.0 buffer solutions [15]. Total dry matter loss (DML) was determined after 30 days of storage by weighing the silo system both before and after ensiling. Total dry matter loss (DML) was determined after 30 days of storage. The DML (%) was calculated as:

\[
\rm DML{{ }}\left( \%  \right) = \left[ {\left( {IDM\;{-}\;FDM} \right)/\;IDM} \right]{\mathrm{ }} \times 100\
\]

Where, IDM (initial dry matter mass) is calculated from the initial as-fed mass and dry matter content of TMR prior to storage, and FDM (final dry matter mass) is calculated from the final as-fed mass and dry matter content at silo opening. The IDM was calculated as:

\[
{\mathrm{IDM \;(gm)}}\;{\mathrm{ =  Initial \;fresh \;mass \;(gm)  \times  Initial \;DM \;fraction,}}
\]

While, FDM was calculated as

\[
{\mathrm{FDM \;(gm)}}\; = \;{\mathrm{Final \;fresh \;mass \;(gm)}}\; \times \;{\mathrm{Final \;DM \;fraction}}{\mathrm{.}}
\]

Ammonia (NH_3_) concentrations were measured using the Conway microdiffusion method [19]. A sealed Conway dish contained 1 ml of supernatant, 1 ml of saturated Na_2_CO_3_, and 1 ml of boric acid, with 1 ml of boric acid placed in the center. After a 24 h incubation period, the central well was titrated with 0.005 N H_2_SO_4_ to calculate the NH_3_ concentration (mM).

Total volatile fatty acids (TVFA) were quantified using steam distillation [20]. In a preconditioned apparatus, 5 ml of sample and 1 ml of 15% H_2_SO_4_ were distilled, capturing VFA in 5 ml of 0.5 N NaOH. The distillate was then titrated with 0.5 N HCl. All the studies utilized a 2 × 4 factorial completely randomized design with five replications (blocks).

### 2.5. In vitro rumen fermentation and gas production analysis

Ground total mixed ration (TMR) anaerobic-stored and aerobic-stored samples were evaluated using an *in vitro* rumen incubation system that included a rumen fluid–buffer mixture, as previously described by Theodorou et al. [21]. In brief, 0.75 gm of each sample was placed into a 125-ml incubation bottle and mixed with 75 ml of inoculum containing 15 ml of rumen fluid and 60 ml of buffer. To ensure anaerobic conditions before incubation, all bottles were flushed with CO_2_, then sealed with rubber stoppers and aluminum crimps. The incubation occurred in a water bath at 39°C for 24 h. Cumulative gas production was monitored at 2, 4, 6, 8, 10, 12, and 24 h using a 50-ml plastic syringe fitted with a needle, and the bottles were manually shaken immediately after each reading. The gas was collected in a reservoir for methane analysis, and methane (CH_4_) concentration was determined via gas chromatography (Shimadzu 8A GC, Shimadzu Corp., Kyoto, Japan) with a flame ionization detector, in accordance with the methodology outlined by Wang et al. [22]. The methane was measured only as a concentration without correcting for headspace pressure, temperature, or water vapor. Thus, the methane results should be viewed primarily as relative treatment differences rather than as absolute standardized yields.

A separate set of bottles containing the same substrates and inoculum was incubated for 24 h to assess rumen fermentation characteristics and *in vitro* digestibility. Following incubation, the contents of the bottles were centrifuged to separate the supernatant from the solid fractions; the supernatant was analyzed for total volatile fatty acid (TVFA) and ammonia (NH_3_) concentrations as previously stated. The solid residue underwent a second-stage digestion in 75 ml of 0.2 N pepsin–HCl for another 24 h. The final residue was vacuum-filtered, dried in an oven at 105°C for 24 h, and subsequently ashed at 500°C for 4 h to assess the residual dry matter (DM) and organic matter (OM). *In vitro* DM digestibility (DMDi) and *in vitro* OM digestibility (OMDi) were calculated as the difference between the initial and residual DM and OM, respectively, following the two-stage process described by Tilley and Terry [23]. All incubations were performed in five replications, and sample allocation was randomized using a complete block design.

### 2.6. Statistical analysis

The collected data were analyzed using factorial statistical models with two fixed factors. The first factor was preservation condition, with anaerobic-stored TMR and aerobic-stored TMR. The second factor was the type of shrimp shell–derived additive, namely control (without additive), shrimp shell, chitin, and chitosan, each applied at 10 gm/kg of fresh TMR. Data on preservation characteristics and *in vitro* rumen fermentation were analyzed using analysis of variance (ANOVA) under a 2 × 4 factorial randomized complete block design. The LSMEANS statement was used for mean separation, and differences among treatment means were compared using Duncan’s multiple range test. Statistical significance was declared at *p* < 0.05, whereas 0.05 ≤ *p* < 0.10 was considered a tendency. All statistical analyses were performed using SAS software version 9.1 (SAS Institute Inc., Cary, NC, USA).

For preservation characteristics, the blocks were defined as different TMR preparation batches to account for possible variation among ingredient batches and sample preparation. For *in vitro* rumen fermentation variables, the blocks were defined as different rumen fluid collection runs, accounting for variation in microbial activity among inoculum collections. In each block, all eight treatment combinations were represented.

## 3. Results

### 3.1. Organoleptic and nutritive quality of preserved TMR

TMR preservation condition clearly affected organoleptic quality, particularly color, aroma, texture, and fungal presence (Table 3). Compared with aerobic-stored TMR, anaerobic-stored TMR had lower organoleptic scores, indicating better visual and sensory quality. The type of shrimp shell–derived additive significantly affected color (*p* < 0.01), aroma (*p* < 0.01), and texture (*p* < 0.01). Among the additives, chitosan produced the most favorable responses, as reflected by the lowest mean scores for color and aroma (both 1.75), whereas the control and shrimp shell treatments had higher scores (2.38–2.50), and chitin showed intermediate values.

**Table 3.** Effects of TMR anaerobic and aerobic-stored inclusion with shrimp shell–derived additives (shrimp shell, chitosan, and chitin) on organoleptic quality scores of TMR.

Items	Treatment (T)	Type of shell-derived (A)				Mean	SEM	*p – value*			
		Control	Shrimp shell	Chitosan	Chitin			T	A	T × A	Block
Colour score	TMR anaerobic-stored	2.00	2.00	1.00	1.75	1.69^χ^	0.122	<0.0001	0.0078	0.1733	0.069
	TMR aerobic-stored	2.75	2.75	2.50	2.75	2.69^y^					
	Mean	2.38^a^	2.38^a^	1.75^b^	2.25^a^						
Aroma score	TMR anaerobic-stored	2.00	2.00	1.00	1.5	1.63^χ^	0.133	<0.0001	0.0003	0.365	0.1561
	TMR aerobic-stored	3.00	3.00	2.50	2.75	2.81^y^					
	Mean	2.50^a^	2.50^a^	1.75^c^	2.13^b^						
Texture score	TMR anaerobic-stored	2.00^b^	2.00^b^	1.00^a^	1.50^a^^b^	1.63^χ^	0.1	<0.0001	0.0081	0.0081	0.001
	TMR aerobic-stored	2.25^b^	2.25^b^	2.25^b^	2.25^b^	2.25^y^					
	Mean	2.13^a^	2.13^a^	1.63^c^	1.88^b^						
Fungal presence score	TMR anaerobic-stored	2.00	2.00	1.00	1.75	1.69^χ^	0.113	<0.0001	0.0546	0.0546	0.0324
	TMR aerobic-stored	2.50	2.50	2.50	2.50	2.50^y^					
	Mean	2.25	2.25	1.75	2.13						

SEM = standard error of the mean, TMR = total mixed ration, T = Preservation treatment, A = Type of shrimp shell-derived additive. Within each variable, different superscript letters indicate significant differences among means (*p* < 0.05).

A significant treatment × additive interaction was observed for texture score (*p* < 0.01). Under anaerobic-stored conditions, chitosan produced the lowest texture score (1.00), indicating non-clumped and non-slimy material, whereas the control and shrimp shell treatments had higher scores (2.00), indicating slight clumping and slime formation. Under aerobic-stored conditions, texture scores were consistently higher across all additive types (2.25), suggesting that differences among additives were less pronounced under continuous oxygen exposure. The fungal presence score was mainly affected by preservation condition (*p* < 0.0001), with lower scores in anaerobically stored TMR than in aerobically stored TMR.

The nutritive value of TMR showed only modest variation among treatments (Table 4); therefore, these data are presented descriptively. In anaerobic-stored TMR, crude protein (CP) ranged from 10.54% to 11.12% DM, with the highest value observed in the chitosan treatment. In aerobic-stored TMR, CP ranged from 10.19% to 10.68% DM, with the highest value in the shrimp shell treatment. Ether extract (EE) was consistently higher in aerobic-stored TMR (6.55–6.88% DM) than in anaerobic-stored TMR (6.13–6.41% DM). Crude fiber (CF) was relatively similar across treatments, ranging from 6.91% to 7.95% DM. Crude ash ranged from 13.80% to 14.49% DM in anaerobic-stored TMR and from 13.12% to 14.30% DM in aerobic-stored TMR.

**Table 4.** Nutritive value of TMR anaerobic and aerobic stored inclusion with shrimp shell–derived additives (shrimp shell, chitosan, and chitin).

Composition	TMR anaerobic-stored				TMR aerobic-stored			
	Control	Shrimp shell	Chitosan	Chitin	Control	Shrimp shell	Chitosan	Chitin
Crude protein (% DM)	10.54	10.67	11.12	10.79	10.19	10.68	10.47	10.31
Ether extract (% DM)	6.13	6.21	6.41	6.33	6.55	6.66	6.78	6.88
Crude fiber (% DM)	7.16	7.25	7	7.95	7.17	7.47	7.7	6.91
Crude ash (% DM)	14.18	14.13	13.8	14.49	13.12	13.77	13.87	14.3
NDF (% CF)	64.74	61.92	62.43	62.62	62.09	55.33	63.71	61.57
ADF (% CF)	48.04	54.45	52.16	48.6	43.56	44.8	44.2	45.65
NDICP (% CP)	7.97	6.08	8.45	8.92	8.62	8.9	8.59	8.61
ADICP (% CP)	4.72	5.39	7.58	6.75	4.45	4.78	4.05	3.61

DM = dry matter, CF = crude fiber, CP = crude protein, NDF = neutral detergent fiber, ADF = acid detergent fiber, NDICP = neutral detergent insoluble crude protein, ADICP = acid detergent insoluble crude protein.

Some variation was more apparent in the fiber-bound fractions. In anaerobic-stored TMR, neutral detergent fiber (NDF) ranged from 61.92% to 64.74% CF, with the lowest value in the shrimp shell treatment. In aerobic-stored TMR, NDF varied more widely, ranging from 55.33% to 63.71% CF, with the lowest value again observed in the shrimp shell treatment. Acid detergent fiber (ADF) ranged from 48.04% to 54.45% CF in anaerobic-stored TMR and from 43.56% to 45.65% CF in aerobic-stored TMR. Neutral detergent insoluble crude protein (NDICP) ranged from 6.08% to 8.92% CP in anaerobic-stored TMR and from 8.59% to 8.90% CP in aerobic-stored TMR. Acid detergent insoluble crude protein (ADICP) in anaerobic-stored TMR ranged from 4.72% to 7.58% CP and reached its highest value in the chitosan treatment, whereas aerobic-stored TMR showed lower ADICP values overall (3.61–4.78% CP).

### 3.2. Preservation characteristics of treated TMR

Preservation condition significantly affected all measured preservation variables (Table 5). Compared with aerobic-stored TMR, anaerobic-stored TMR had lower dry matter loss (DML), lower pH, higher total volatile fatty acids (TVFA), and lower NH_3_ concentration (*p* < 0.01), indicating a more preserved anaerobic environment.

**Table 5.** Effects of TMR anaerobic and aerobic-stored inclusion with shrimp shell–derived additives (shrimp shell, chitosan, and chitin) on fermentation characteristics of TMR.

Items	Treatment (T)	Type of shell-derived (A)				Mean	SEM	*p* – *value*			
		Control	Shrimp shell	Chitosan	Chitin			T	A	T × A	Block
DML (%)	TMR anaerobic-stored	3.92^a^	4.06^ab^	3.50^a^	4.04^ab^	3.88^χ^	0.622	<0.0001	<0.0001	<0.0001	0.9869
	TMR aerobic-stored	11.69^e^	11.21^e^	9.46^c^	10.38^d^	10.69^y^					
	Mean	7.81^a^	7.64^a^	6.48^c^	7.21^b^						
pH	TMR anaerobic-stored	6.03^b^	6.00^b^	5.20^a^	5.97^b^	5.80^χ^	0.057	<0.0001	<0.0001	<0.0001	0.1383
	TMR aerobic-stored	6.20^c^	6.20^c^	6.20^c^	6.20^c^	6.20^y^					
	Mean	6.12^a^	6.10^a^	5.70^b^	6.09^a^						
TVFA (mM/l)	TMR anaerobic-stored	13.17	13.19	13.15	13.18	13.17^y^	0.995	<0.0001	0.5213	0.6904	0.8617
	TMR aerobic-stored	2.09	2.10	2.09	2.08	2.09^χ^					
	Mean	7.63	7.65	7.62	7.63						
NH_3_ (mM/l)	TMR anaerobic-stored	4.38^a^	4.71^a^	4.50^a^	4.47^a^	4.52^χ^	0.211	<0.0001	0.0277	0.0285	0.9542
	TMR aerobic-stored	6.49^b^	6.58^b^	7.35^c^	6.58^b^	6.75^y^					
	Mean	5.44^b^	5.65^ab^	5.93^a^	5.53^b^						

DML = dry matter loss, TVFA = total volatile fatty acids, NH_3_ = ammonia, SEM = standard error of mean, T = Preservation treatment, A = Type of shrimp shell-derived additive. Within each variable, different superscript letters indicate significant differences among means (*p* < 0.05).

Dry matter loss was affected by preservation condition, additive type, and their interaction (*p* < 0.01). Under anaerobic storage conditions, mean DML was low (3.88%; range, 3.50%–4.06%), with chitosan showing the lowest value (3.50%). Under aerobic storage conditions, DML was markedly higher (mean 10.69%), ranging from 9.46% to 11.69%. Even under aerobic storage, chitosan reduced DML to 9.46%, compared with 11.69% in the control and 11.21% in the shrimp shell treatment, while chitin was intermediate (10.38%).

The pH values were also affected by preservation condition, additive type, and their interaction (*p* < 0.01). Anaerobic-stored TMR had a lower mean pH than aerobic-stored TMR (5.80 vs. 6.20), and this reduction was mainly associated with chitosan, which reached the lowest pH value (5.20) under anaerobic conditions. In contrast, the control, shrimp shell, and chitin treatments under anaerobic conditions remained within a narrow range of 5.97–6.03, while all aerobic-stored treatments had the same pH value (6.20). Although anaerobic storage reduced pH relative to aerobic storage, all pH values remained above the range generally associated with conventional silage fermentation, indicating that acidification was limited under the present TMR conditions.

Total volatile fatty acids were strongly affected by preservation condition (*p* < 0.01) but not by additive type or the treatment × additive interaction (*p* > 0.05). Anaerobic-stored TMR had substantially higher TVFA than aerobic-stored TMR (13.17 vs. 2.09 mM/l), confirming that fermentation activity was greater under anaerobic than aerobic conditions.

Ammonia concentration was lower in anaerobically stored than in aerobically stored TMR (4.52 vs. 6.75 mM/l; *p* < 0.01) and was also affected by additive type and the treatment × additive interaction (*p* < 0.05). Under anaerobic storage conditions, NH_3_ concentrations were similar among additives (4.38–4.71 mM/l). In contrast, under aerobic storage conditions, chitosan increased NH_3_ concentration to 7.35 mM/l, whereas the control, shrimp shell, and chitin treatments ranged from 6.49 to 6.58 mM/l. This higher NH_3_ value in aerobic-stored TMR with chitosan was the main contributor to the significant interaction and is examined further in the Discussion. No block effect was detected for preservation variables (*p* > 0.05).

### 3.3. Effects of preservation condition and shrimp shell–derived additives on in vitro rumen fermentation

Preservation condition did not significantly affect *in vitro* dry matter digestibility (DMDi), *in vitro* organic matter digestibility (OMDi), or NH_3_ concentration (*p* > 0.05), but it significantly affected TVFA (*p* < 0.01) (Table 6). Anaerobic-stored TMR produced slightly higher TVFA than aerobic-stored TMR (121.91 vs. 118.35 mM).

**Table 6.** Effects of TMR anaerobic and aerobic-stored inclusion with shrimp shell–derived additives (shrimp shell, chitosan, and chitin) on *in vitro* rumen fermentation profiles.

Items	Treatment (T)	Type of shell-derived (A)				Mean	SEM	*p* – *value*			
		Control	Shrimp shell	Chitosan	Chitin			T	A	T × A	Block
DMDi (%)	TMR anaerobic-stored	48.39	48.54	57.47	53.82	52.06	0.721	0.8439	0.0015	0.2065	0.3674
	TMR aerobic-stored	50.17	52.46	55.75	50.84	52.3					
	Mean	49.28^d^	50.50^c^	56.61^a^	52.33^b^						
OMDi (%)	TMR anaerobic-stored	49.08	49.51	57.39	54.67	52.66	0.726	0.1959	0.0006	0.3194	0.563
	TMR aerobic-stored	51.75	53.89	59.21	52.55	54.35					
	Mean	50.42^d^	51.70^c^	58.30^a^	53.61^b^						
TVFA (mM)	TMR anaerobic-stored	124.50^d^	123.39^d^	115.42^b^	124.31^d^	121.91^y^	0.822	<0.0001	<0.0001	0.0392	0.0426
	TMR aerobic-stored	119.25^c^	120.51^c^	111.14^a^	122.52^c^^d^	118.35^χ^					
	Mean	121.88^b^	121.95^b^	113.28^c^	123.41^a^						
NH_3_ (mM/l)	TMR anaerobic-stored	12.28	11.58	11.03	11.58	11.62	0.273	0.135	0.0256	0.0709	0.0808
	TMR aerobic-stored	10.66	13.13	9.48	10.25	10.88					
	Mean	11.47^b^	12.36^a^	10.25^c^	10.92^b^^c^						

DMDi = *in vitro* dry matter digestibility, OMDi = *in vitro* organic matter digestibility, TVFA = total volatile fatty acids, NH_3_ = ammonia, SEM = standard error of mean, T = Preservation treatment, A = Type of shrimp shell-derived additive. Within each variable, different superscript letters indicate significant differences among means (*p* < 0.05).

The additive type significantly affected DMDi (*p* < 0.01) and OMDi (*p* < 0.01). Chitosan yielded the highest mean digestibility values for both DMDi (56.61%) and OMDi (58.30%), followed by chitin (52.33% and 53.61%), shrimp shell (50.50% and 51.70%), and the control (49.28% and 50.42%). No preservation condition × additive interaction was detected for DMDi or OMDi (*p* > 0.05), indicating that the positive effect of chitosan on digestibility was consistent across both preservation conditions.

Fermentation end products were also affected by treatment. TVFA was influenced by preservation condition, additive type, and their interaction (*p* < 0.01, *p* < 0.01, and *p* < 0.05, respectively), with a minor block effect (*p* < 0.05). In both preservation conditions, chitosan produced the lowest TVFA values, namely 115.42 mM in anaerobic-stored TMR and 111.14 mM in aerobic-stored TMR, whereas the other additives generally ranged from 119 to 125 mM. Additive type affected NH_3_ concentration (*p* < 0.05), with a tendency for interaction (*p* < 0.10). Overall, shrimp shell produced the highest NH_3_ concentration (12.36 mM/l), whereas chitosan produced the lowest (10.25 mM/l).

### 3.4. Methane concentration at 24 h and total gas production kinetics

**Figure 1.**
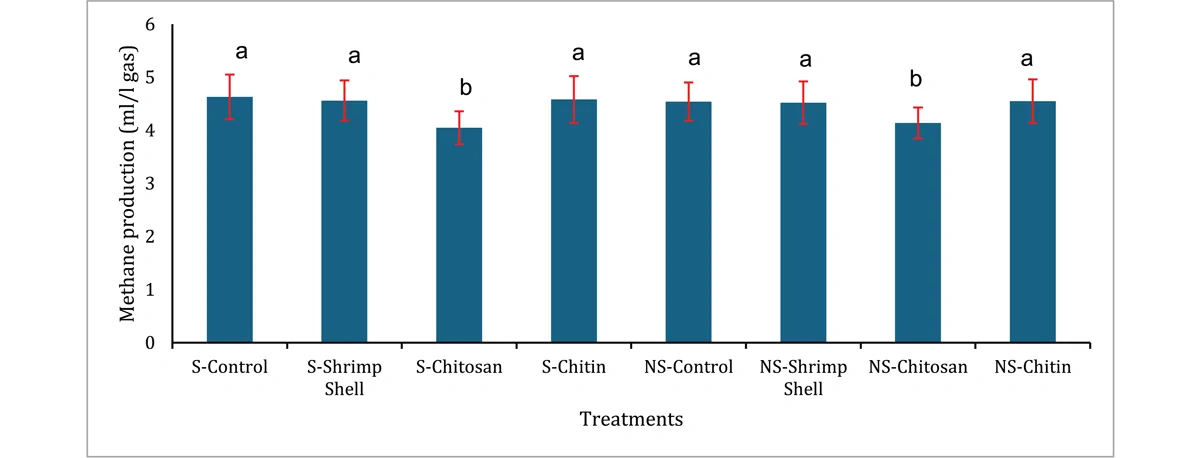
*In vitro* methane (CH_4_) production (ml/l gas) of TMR stored anaerobically and aerobic-stored inclusion with shrimp shell–derived additives (shrimp shell, chitosan, and chitin). Treatments: S-Control, TMR anaerobic-stored without additive; S-Shrimp shell, TMR anaerobic-stored + shrimp shell additive; S-Chitosan, TMR anaerobic-stored + chitosan; S-Chitin, TMR anaerobic-stored + chitin; NS-Control, TMR aerobic-stored without additive; NS-Shrimp shell, TMR aerobic-stored + shrimp shell additive; NS-Chitosan, TMR aerobic-stored + chitosan; NS-Chitin, TMR aerobic-stored + chitin. Error bars indicate the standard error of the mean (SEM). Different superscript letters (a–b) indicate significant differences among additive types (*p <* 0.05).

Methane concentration in the fermentation gas at 24 h varied with the type of additive (Figure 1). Under both anaerobic- and aerobic-stored TMR, chitosan consistently yielded the lowest CH_4_ concentration (*p* < 0.05), whereas the control, shrimp shell, and chitin treatments showed higher, relatively similar values. No time-course methane analysis was conducted; therefore, only the 24 h methane concentration is presented and interpreted.

**Figure 2.**
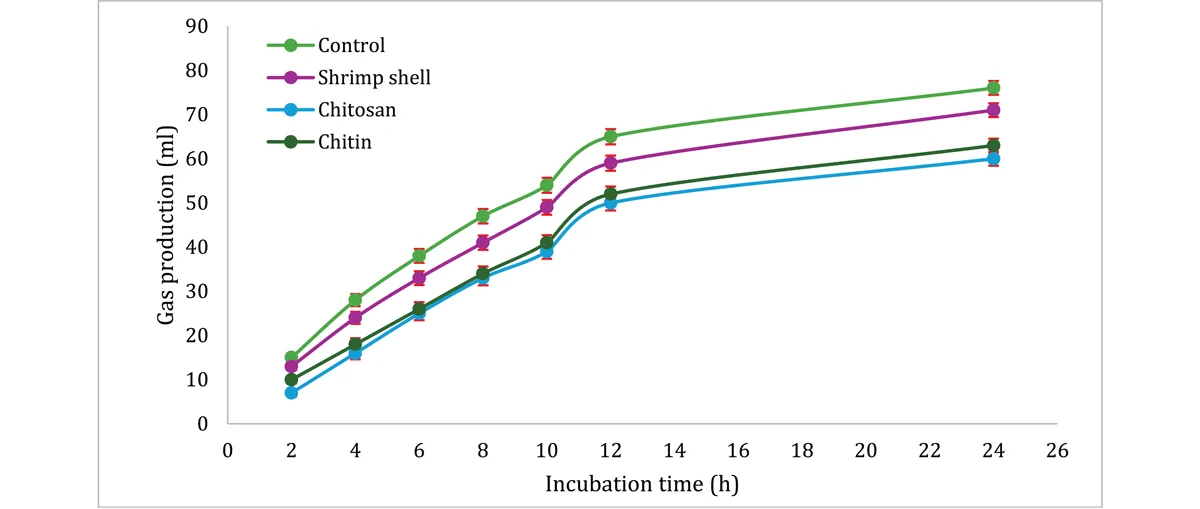
*In vitro* rumen production kinetics of total gas (ml) from TMR anaerobically stored with various shrimp shell-derived additives after 2, 4, 6, 8, 10, 12, and 24 h of incubation.

**Figure 3.**
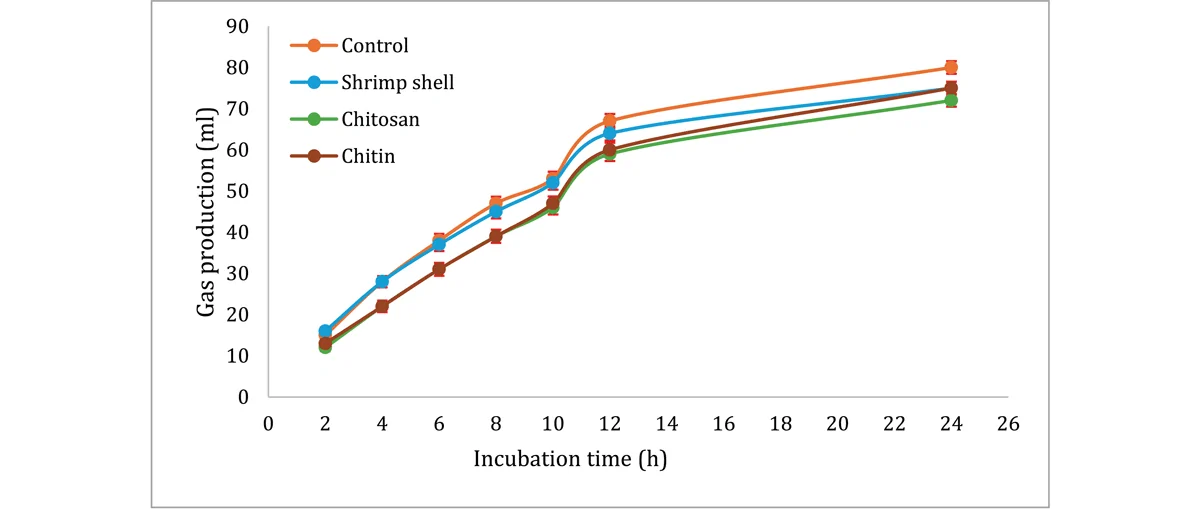
*In vitro* rumen production kinetics of total gas (ml) from TMR, aerobically stored with various shrimp shell-derived additives after 2, 4, 6, 8, 10, 12, and 24 h of incubation.

Total gas production increased with incubation time in all treatments (Figures 2, 3). In anaerobic-stored TMR, the control treatment consistently produced the highest gas volume, followed by shrimp shell, whereas chitosan produced the lowest cumulative gas volume throughout incubation. Chitin also reduced gas production relative to the control, although less markedly than chitosan. A similar ranking was observed in aerobic-stored TMR, where chitosan again produced the lowest total gas volume, while the control remained the highest. These figures are presented descriptively to illustrate fermentation kinetics, whereas treatment interpretation was based primarily on the 24 h response variables.

## 4. Discussion

The enhanced sensory characteristics of anaerobically stored TMR with chitosan are indicated by significant impacts on color, aroma, texture, and fungal presence. Chitosan received the best sensory-quality ratings due to its solubility and polycationic properties. A previous study reported that chitosan reduced the growth of spoilage microorganisms in anaerobically stored TMR, such as Clostridia and yeast, while increasing beneficial lactic acid bacteria, which help maintain the quality and sensory properties of the anaerobic preservation [14, 24, 25]. Crude shrimp shell had lower acceptability due to its complex composition, which adds marine flavors and affects acidification. The presence of residual proteins and lipids in shrimp shells can contribute to off-flavors and odors. Chitin showed an intermediate response, which might be attributed to its lower solubility and reduced availability of reactive amino groups compared with chitosan, thereby limiting its functional activity. It has been reported that chitin possesses antimicrobial properties, but its effectiveness is generally lower than that of chitosan due to physicochemical limitations [26].

The nutritive value data exhibited only slight variation among treatments and were presented descriptively. However, certain patterns deserve closer examination. The slightly higher crude protein (CP) in anaerobically stored TMR, especially with chitosan, may indicate lower nitrogen loss during storage. Previous studies have reported that chitosan supplementation at 1–1.5% preserves the nutritional quality of silage by reducing fermentation losses without affecting its physical quality [24, 27]. The values for ether extract (EE) and crude fiber (CF) were similar across all treatments. Ash content varied modestly, consistent with shrimp shell minerals like CaCO_3_, without significant dilution or concentration changes. Shrimp shells contain chitin, proteins, and calcium carbonate. The presence of CaCO_3_ is crucial for the structural integrity and rigidity of the shells [28]. Furthermore, the advantages of using shrimp shell additives, especially chitosan, primarily relate to enhanced preservation and fermentation control rather than notable alterations in nutritional value.

The presence of neutral detergent insoluble crude protein (NDICP) and acid detergent insoluble crude protein (ADICP) suggests that shrimp shells, along with chitin and chitosan, contain a protein matrix. Chitin and chitosan are closely related natural polysaccharides with significant differences in their chemical structures and properties. Chitin is a long-chain polymer of N-acetylglucosamine, which forms a polysaccharide skeleton strengthened and modified by a protein matrix containing nitrogen [29, 30]. Chitosan, derived from chitin through partial deacetylation, possesses a highly functional amino group (-NH_2_) at the C-2 position, distinguishing it from cellulose, which has a hydroxyl group (-OH) at the same position [31].

The current study demonstrated that TMR stored anaerobically showed reduced dry matter loss, lower pH, and lower NH_3_ levels, while exhibiting higher total volatile fatty acids (TVFA), indicating a more stable biochemical environment compared to aerobic conditions. Chitosan effectively reduced DM loss in both conditions but showed the highest benefit in anaerobically stored conditions. A previous study reported that chitosan has antimicrobial properties that effectively reduce mold and yeast counts in anaerobic preservation [32]. Moreover, another study reported that chitosan decreased fermentative losses and improved DM recovery in rehydrated corn silage [33]. This improved fermentation profile is linked to enhanced anaerobic preservation stability and quality. However, total volatile fatty acids (TVFA) increased significantly during the anaerobic process, as expected due to active fermentation.

During ensiling, the additives showed significantly different effects on pH. Notably, chitosan had a much lower pH than shrimp shell or chitin. According to a previous study, chitosan increased the concentrations of acetic and propionic acids in silage [33], resulting in a lower pH in anaerobically stored silage. In contrast, several studies indicate that chitosan increases pH during anaerobic preservation, as observed in chitosan-treated alfalfa silage [22], sugarcane silage [34], and Mombasa grass silage [34]. This contradiction likely arises from variations in forage type, water-soluble carbohydrate content, buffering capacity, moisture level, and chitosan properties, such as molecular weight and degree of deacetylation, which may explain the inconsistent results across studies. Previous studies have reported that silage with high water-soluble carbohydrate (WSC) content tends to have a lower pH due to more efficient fermentation [35]. In contrast, the balance between WSC and protein is crucial, as silage with high protein and low WSC content tends to have a higher pH, which may be associated with increased activity of butyric acid–producing bacteria and higher protein degradation [36].

The findings regarding ammonia (NH_3_) suggest that anaerobic conditions reduce NH_3_ levels, thereby reducing proteolysis, while chitosan increases NH_3_ levels compared to other additives. A previous study reported that chitosan increases NH_3_ levels in anaerobic preservation [32]. Moreover, when chitosan is placed in dilute acid solutions (pH < 6.3), the amino groups (NH_2_) become protonated, transforming into the soluble form (NH_3_^+^) [37]. Thus, the elevated NH_3_ levels observed in the chitosan treatment are likely not due to increased protein degradation during fermentation, but rather associated with the chemical behaviour of chitosan itself. However, in some anaerobic preservation studies, where chitosan does not significantly impact NH_3_ levels [33, 34].

In the present study, the absence of a preservation-condition effect on DMDi and OMDi indicates that anaerobic storage did not significantly alter the digestibility of the substrate compared with aerobic storage, even though it altered the preservation characteristic. Furthermore, previous studies indicate improved nutrient digestibility, possibly due to reduced protozoal predation on bacteria, leading to more efficient bacterial protein synthesis [38]. However, some studies report that chitosan decreased digestibility at higher chitosan levels, potentially due to its antimicrobial effects, altering microbial fermentation patterns [10, 39, 40]. The inconsistent effects of chitosan on DMDi and OMDi stem from its dose-dependent antimicrobial activity, as well as variations in forage-to-concentrate ratio, substrate characteristics, rumen pH, and chitosan (degree of deacetylation and molecular weights). For instance, higher doses (e.g., 8–12 gm/kg DM) reduced DMDi and OMDi in some cases, possibly due to excessive antimicrobial activity that disrupted rumen microbial balance [39, 40]. However, moderate doses (e.g. 0.6 gm/kg DM) consistently improved digestibility [41].

Current research indicates that total volatile fatty acids (TVFA) decrease with the use of chitosan. This effect may arise from chitosan’s ability to manipulate rumen fermentation, which could lead to reduced hydrogen production associated with acetate and potentially lower concentration of gas and methane (CH_4_) [42]. In addition, another study reported that supplementation with chitosan reduced acetate proportions in both *in vitro* and *in vivo* experiments, leading to a lower acetate-to-propionate (C_2_:C_3_) ratio [7, 41, 43]. The increase in propionate is attributed to changes in the rumen microbial community, favouring propionate-producing bacteria such as Prevotella [7, 44]. Supporting evidence from studies on total mixed rations (TMR) and anaerobic preservation suggests that chitosan reduces protozoa, total gas production, methane levels, and the proportion of acetate, while increasing propionate levels [11]. Furthermore, an *in vivo* metagenomic study corroborated these findings, demonstrating decreased protozoa and a shift toward a lower acetate-to-propionate ratio with chitosan supplementation [8].

The present study showed that chitosan producing the lowest ammonia (NH_3_) concentration in the rumen *in vitro*. In addition, a previous study reported that chitosan decreased NH_3_ concentration in the rumen *in vitro*, likely due to its antimicrobial properties, which inhibit proteolytic bacteria responsible for protein degradation and NH_3_ production [25, 45]. By reducing protozoal populations, chitosan indirectly lowers NH_3_ release from protein breakdown [25, 32, 33]. In addition, chitosan alters the rumen fermentation profile by increasing propionate production and reducing the proportions of acetate and butyrate. This shift may reduce nitrogen recycling and NH_3_ accumulation in the rumen [14, 32, 33]. However, the impact of chitosan on ammonia concentration is dose-dependent, as evidenced by a study showing that intermediate doses (e.g., 800 mg/kg DM) appear to optimize reductions in ammonia while maintaining other fermentation parameters [46, 47].

Chitosan affects the kinetics of gas production by reducing both the rate and the total amount of gas produced. In a previous study, it was reported that the level of chitosan (1.5% to 3.0%) can significantly reduce asymptotic gas production [24, 25]. In addition, chitosan has been shown to reduce methane (CH_4_) production, likely due to its antimicrobial effects on methanogens [39, 43]. The evidence from a previous study shows that chitosan decreases protozoal activity and methanogen populations, which are key contributors to methane production in the rumen [42, 48]. Furthermore, chitosan alters volatile fatty acid (VFA) profiles by increasing propionate levels and reducing acetate proportions, thereby lowering the acetate-to-propionate ratio [8, 9, 48]. This shift is significant because propionate production competes with methanogenesis for hydrogen, thereby reducing methane emissions [9, 42]. However, shrimp shell and chitin showed no *in vitro* rumen-modulatory effects due to their low solubility and limited bioactivity at the tested dose. Crude shrimp shells contain inert minerals, proteins, and pigments, which may hinder their bioactivity and solubility [49, 50].

## 5. Conclusions

Shrimp shell–derived chitosan at 1% of fresh matter (10 gm/kg fresh TMR) was the most effective additive among those tested for improving anaerobic TMR preservation and modulating in vitro rumen fermentation. Compared with raw shrimp shell and chitin, chitosan was associated with better organoleptic quality, lower pH in anaerobic TMR, reduced dry matter loss, higher dry and organic matter digestibility, and lower methane concentration. These findings indicate potential benefits for farmers and feed manufacturers and highlight the value of shrimp-processing waste in a circular economy. Further research is needed to confirm these results through *in vivo* trials and to evaluate factors such as feed efficiency, economic feasibility, and methane mitigation impacts.

## Data Availability

The data presented in this study are available from the corresponding author upon reasonable request.

## List of abbreviations

ADF, acid detergent fiber; ADICP, acid detergent insoluble crude protein; AOAC, Association of Official Analytical Chemists; CH_4_, methane; CP, crude protein; DMDi, *in vitro* dry matter digestibility; DM, dry matter; DML, dry matter loss; EE, ether extract; GC, gas chromatography; HDPE, high-density polyethylene; IRB, Institutional Review Board; LAB, lactic acid bacteria; N, nitrogen; NDF, neutral detergent fiber; NDICP, neutral detergent insoluble crude protein; NH_3_, ammonia; OM, organic matter; OMDi, *in vitro* organic matter digestibility; TMR, total mixed ration; TVFA, total volatile fatty acids.
